# Dexmedetomidine Injection during Strabismus Surgery Reduces Emergence Agitation without Increasing the Oculocardiac Reflex in Children: A Randomized Controlled Trial

**DOI:** 10.1371/journal.pone.0162785

**Published:** 2016-09-12

**Authors:** In-Ae Song, Kwang-Suk Seo, Ah-Young Oh, Ji-Seok Baik, Jin Hee Kim, Jung- Won Hwang, Young-Tae Jeon

**Affiliations:** 1 Department of Anesthesiology and Pain Medicine, Seoul National University Bundang Hospital, Seongnam, Korea; 2 Department of Dental Anesthesiology, Seoul National University School of Dentistry, Seoul, Korea; 3 Department of Anesthesiology and Pain Medicine, Seoul National University College of Medicine, Seoul, Korea; 4 Department of Anesthesiology and Pain Medicine, Pusan National University Hospital, Busan, Korea; University of Bari, ITALY

## Abstract

**Objective:**

Dexmedetomidine is known to reduce the incidence of emergence agitation, which is a common complication after inhalational anesthesia like sevoflurane or desflurane in children. However, the dose of dexmedetomidine used for this purpose is reported variously and the most effective dose is not known. In this study, we tried to find the most effective dose of dexmedetomidine to reduce the incidence of emergence agitation in children undergoing strabismus surgery without the complications like oculocardiac reflex (OCR) or postoperative vomiting.

**Methods:**

We randomized 103 pediatric patients aged 2–6 years and undergoing elective strabismus surgery into four groups. Anesthesia was induced with sevoflurane and maintained with desflurane. At the start of induction, dexmedetomidine, delivered at 0.25, 0.5, or 1 μg/kg, or saline was infused intravenously in the D0.25, D0.5, D1 groups, respectively. The primary outcome measure was the incidence of emergence agitation and the secondary outcome measure was the incidence of intraoperative OCR, postoperative vomiting, and desaturation events.

**Results:**

The incidence of emergence agitation was 60, 48, 44, and 21% (*P* = 0.005) and the incidence of intraoperative OCR was 36, 36, 36, and 37% (*P* = 0.988) in the control, D0.25, D0.5, and D1 groups, respectively. And, postoperative vomiting rate and desaturation events were low in the all groups.

**Conclusion:**

Dexmedetomidine decreased the incidence of emergence agitation without increasing intraoperative oculocardiac reflex. Dexmedetomidine delivered at 1 μg/kg was more effective at reducing emergence agitation than lower doses in children undergoing strabismus surgery under desflurane anesthesia.

**Trial Registration:**

Clinical Research Information Service KCT0000141

## Introduction

Desflurane is very useful for pediatric strabismus surgery because the emergence from anesthesia with desflurane is more rapid than other potent inhalation gases. However, both desflurane and strabismus surgery predispose children’s emergence agitation (EA), which might cause patients’ fall-down or self-injury and requires intensive cautions of caregivers and families [1, 2].

EA might be preventable with the perioperative use of dexmedetomidine, a selective α-2-adrenergic agonist with sedative, anxiolytic, and analgesic effects with minimal respiratory depression in the previous studies [3–6]. Its cardiovascular effect is modest, predictable, and dose-dependent [7]. The use of dexmedetomidine in patients has been investigated extensively and it has been used as a premedication to reduce preoperative anxiety[8], as an adjunct to anesthesia [9] for sedation in intensive care units [10], and for procedural sedation [11], without major complications. It also reduced postoperative vomiting (POV) after pediatric strabismus surgery as well as clonidine [12].

However dexmedetomidine might aggravate oculocardiac reflex (OCR) during strabismus surgery because it sometimes caused bradycardia. There has been no study about association of the incidence of OCR and use of perioperative dexmedetomidine.

In this study, we evaluated the effect of dexmedetomidine on EA and examined the dose of dexmedetomidine that best reduced EA. We also evaluated the effect of dexmedetomidine on OCR as the secondary outcome variable.

## Methods

Approval for this study was obtained from the institutional review board of Seoul National University Bundang Hospital, Seongnam, Korea (Chairperson Prof K.C.Park) on 18 April 2011 (B-1103/124-008). The study was registered with the clinical research information service (CRiS), Republic of Korea (URL: https://cris.nih.go.kr/cris/index.jsp), and the number of registration was KCT0000141. This study was conducted according to the principles expressed in the Declaration of Helsinki with written informed parental consents.

The participants was ASA class I pediatric patients, aged 2–6 years had used outpatient surgery services in Seoul National University Bundang Hospital for undergoing elective strabismus surgery, and the complete date range for participant recruitment and follow-up was from February 28, 2013, to February 10, 2014.

The study randomly allocated 103 participants to one of four groups: saline (control) or dexmedetomidine 0.25 (D0.25), 0.5 (D0.5), or 1.0 (D1) μg/kg. Random numbers were generated by a person not involved in the study using a computer-generated randomization code (Random Allocation Software, ver. 1.0; M. Saghaei, Isfahan University of Medical Sciences, Isfahan, Iran). Patients with a known allergy to the drugs to be used or with a developmental delay or neurological disease were excluded from the study. Using a 4-point scale, preoperative agitation was evaluated in the reception area and again in the operating room before applying the mask to the patient. In the operating room, the patient’s electrocardiograph, non-invasive arterial pressure, pulse oximetry, and end-tidal CO_2_ were monitored. Without premedication, the patients were induced by the inhalation of sevoflurane and 60% N_2_O in the presence of one of their parents. After the loss of consciousness, an intravenous line was inserted and the study drug (saline or dexmedetomidine) was infused over 10 min. The study drugs were prepared by an anesthesiologist who did not participate in the care of the patients to the same volume (10 ml) using saline. After a few minutes of mask ventilation with 8% sevoflurane and 60% N_2_O, a laryngeal mask airway (LMA) was inserted and anesthesia was maintained with 8–10% desflurane and 60% N_2_O. The desflurane concentration was adjusted to maintain the blood pressure and heart rate within 20% of the preoperative values. Neuromuscular blockers were not used. At the end of surgery, the LMA was removed in the operating room when the patient could breathe spontaneously and the time from the cessation of desflurane to removal of the LMA was recorded. The operations were done by a single experienced senior surgeon. The definition of OCR was a more than 20% decrease in heart rate from the baseline HR, or the appearance of bradyarrhythmias [13]. The lowest heart rate during the operation was recorded and a decrease in heart rate of > 20% from the baseline value, which was measured immediately before muscle we asked the surgeon to release the traction. Atropine at 0.01 mg/kg was used intravenously only for severe (< 60/min) or persistent bradycardia.

In the post-anesthesia care unit (PACU), EA and pain were evaluated continuously and recorded at 5-min intervals. EA was evaluated using a 4-point scale: 1 = calm; 2 = not calm, but could be calmed easily; 3 = not easily calmed, moderately agitated, or restless; and 4 = combative, excited, or disoriented [14, 15]. Patients with a score ≥ 3 were regarded as having EA [15, 16]. The Pediatric Anesthesia Emergence Delirium (PAED) scale was also checked and patients with a PAED score ≥ 10 were regarded as having EA[17]. Pain was evaluated using the pediatric Face, Legs, Activity, Cry, and Consolability (FLACC) pain scale [18, 19]. The experienced anesthesiologists evaluated the OCR, the severity of EA and pain using the scale described above, were blinded to the patients’ groups. They were standing at the patients’ bedside till they started to expressed the EA or pain, and treat them with fentanyl at 1 μg/kg immediately when they show severe agitation (EA score 4), or a FLACC pain score ≥ 6 [18]. All of patients were encouraged to have 12.8mg/kg of acetaminophen suspension. Metoclopramide 0.1mg/kg IV were administered for patients with nausea and vomiting. Additionally 25mg of pethidine and 1mg/kg of ketorolac were prepared in case agitation or pain was relapsed after fentanyl’s effect disappeared.

All setting for anesthesia and perioperative treatment in the control group of this study was as same as routine children’s strabismus surgery before the study.

The durations of surgery and anesthesia, PACU stay time, and the time from stopping the inhalational agents to removing the LMA were also recorded. The events such as desaturation, bradycardia, nausea and vomiting, deep sedation in PACU were recorded.

### Statistical Analysis

Group sample sizes of 28 was calculated to detect a 40% reduction of the incidence of severe agitation with dexmedetomidine infusion from 57% in control group (normal saline infusion) with 0.05 of alpha and 0.80 of beta using Power Analysis and Sample Size software (PASS) 2013 (NCSS, LLC, Kaysville, Utah, USA) and was added with 10% of dropout rate [3]. The results were evaluated using IBM SPSS Statistics ver. 21 (IBM Corp., Armonk, NY, USA). Age, weight, height, the durations of surgery and anesthesia, time to LMA removal, and recovery time were compared among the groups using a one-way analysis of variance (ANOVA). Heart rate and systolic blood pressure were analyzed using an ANOVA and Bonferroni *post hoc* multiple comparison analysis among the groups. The independence of sex, previous surgical history, and number of muscles operated on were compared using Pearson’s chi-square test or Fisher’s exact test, and the trends in dichotomous data for agitation, fentanyl use, and OCR according to the dexmedetomidine dose were analyzed using a linear-by-linear association and chi-square test. Our analysis included the Kruskal-Wallis test and median tests for nonparametric or ordinal data of preoperative agitation, PAED scale. Nonparametric or ordinal data are presented as the median (25–75% interquartile range); continuous data are presented as the mean and standard deviation. *P*-values < 0.05 were considered significant.

## Results

**Fig 1:**
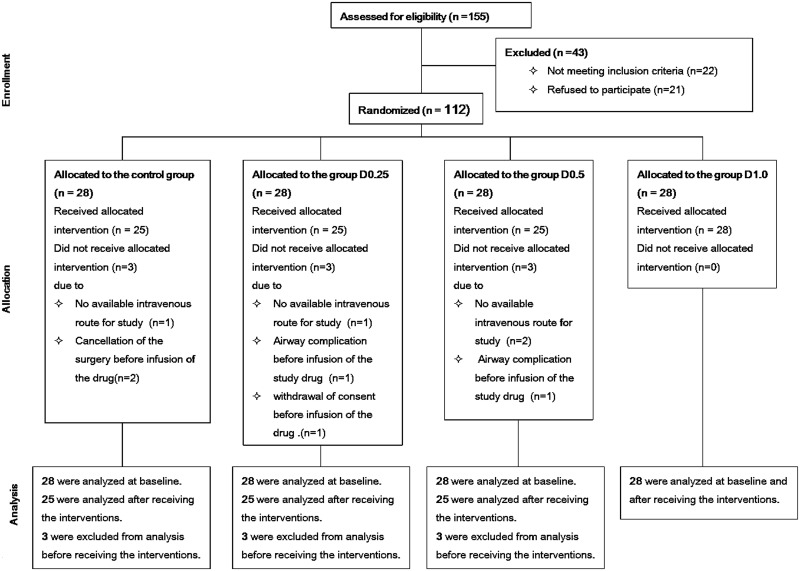
CONSORT flow diagram.

In total, 155 patients were evaluated for enrollment; of these, 22 did not meet the inclusion criteria and 21 refused to participate. As a result, 112 patients were enrolled and randomized to one of the four groups. After enrollment, 9 patients were excluded from the analysis due to violation of the study protocol; thus, 103 patients were analyzed (Fig 1).

The baseline characteristics (age, sex, weight, height, and proportion of patients with a previous operation) for all randomized patients did not differ significantly among the groups (Table 1), and there was no significant difference among procedural characteristics (preoperative agitation score, durations of surgery and anesthesia, number of muscles operated on, time from cessation of anesthetic to LMA removal, and PACU stay time) of the groups including only the patients who received the intervention, respectively(Table 2).

**Table 1:** Baseline characteristics of all randomized patients.

Variable	Control	D 0.25	D 0.5	D 1.0	P-value
**N**	**28**	**28**	**28**	**28**	
**Age (yr)**	3.8 (1.5)	4.3 (1.7)	4.5 (1.3)	4.6 (1.3)	0.130
**Sex (male)**	14 (50%)	14 (50%)	10 (36%)	16 (57.1%)	0.446
**Weight (kg)**	18.1 (4.2)	18.4 (4.5)	19.1 (4.8)	19.7(5.1)	0.592
**Height (cm)**	104.2 (11.7)	107.6 (11.5)	108.2 (11.7)	111.5 (9.9)	0.140
**Previous operation history**	7 (25%)	2 (7.1%)	4 (14.2%)	6 (21.4%)	0.298

Values are mean (SD) or numbers (%).

**Table 2:** Procedural characteristics including only the patients who received the intervention.

Variable	Control	D 0.25	D 0.5	D 1.0	P-value
**N**	**25**	**25**	**25**	**28**	
**Preoperative agitation score (reception)**					0.197
1	18 (72%)	18 (72%)	20 (80%)	23 (82.1%)	
2	5 (20%)	7 (28%)	4 (16%)	5 (17.8%)	
3	2 (8%)	0 (0%)	1 (4%)	0 (0%)	
4	0 (0%)	0 (0%)	0 (0%)	0 (0%)	
**Preoperative agitation score (mask)**					0.081
1	13 (52%)	14 (56%)	18 (72%)	20 (71.4%)	
2	6 (24%)	7 (28%)	6 (24%)	3 (10.7%)	
3	4 (16%)	3 (12%)	1 (4%)	5 (17.8%)	
4	2 (8%)	1 (4%)	0 (0%)	0 (0%)	
**Duration of surgery (min)**	18.8 (7.0)	16.2 (4.4)	15.5 (4.2)	17.8 (7.7)	0.218
**Duration of anesthesia (min)**	30.6 (9.1)	27.9 (4.5)	26.4 (4.5)	28.8 (8.0)	0.207
**Number of muscles operated**					0.611
1	2 (8%)	1 (4%)	4 (16%)	2 (7.1%)	
2	21 (84%)	24 (96%)	21 (84%)	25 (89.2%)	
3	1 (4%)	0 (0%)	0 (0%)	0 (0%)	
4	1 (4%)	0 (0%)	0 (0%)	1 (3.5%)	
**Time to LMA removal (min)**	4.1 (2.1)	4.1 (2.0)	5.6 (3.9)	4.5 (2.1)	0.160
**PACU stay time (min)**	20.4 (9.4)	19.6 (5.3)	23.6 (10.1)	21.0 (7.3)	0.375

Values are mean (SD) or numbers (%).

**Fig 2:**
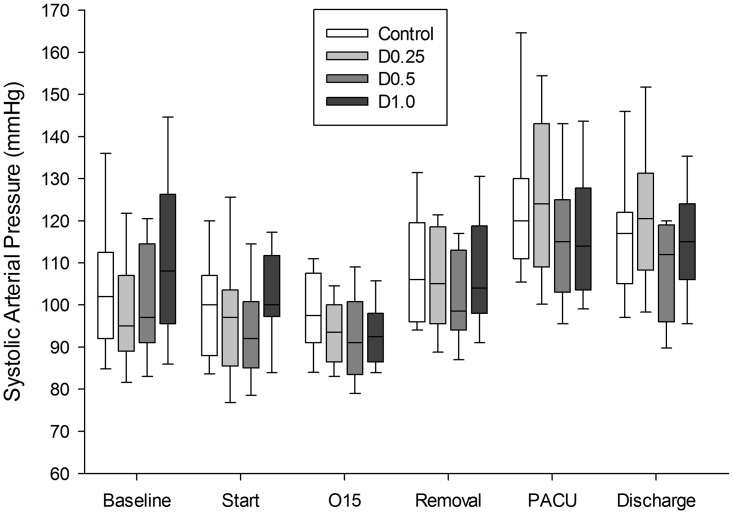
Change in systolic arterial pressure (SAP) during the study period. The horizontal line within the box is median SAP during the study period. The edges of the box and the whiskers indicate 25–75% and 5–95% range respectively. The mean values of SAP were not statistically different during operation and at PACU among the groups. Baseline = before induction; Start = start of operation; O15 = 15 min after start of operation, Removal = LMA removal, PACU = at admission to post-anesthesia care unit; Discharge = PACU discharge.

The incidence of the OCR during surgery was 36, 36, 36, and 37% in the control, D0.25, D0.5, and D1 groups, respectively (*P* = 0.988) and no patient needed atropine to correct bradycardia during the operation. The lowest heart rate during the operation did not differ among the groups. The mean systolic arterial pressure was not significantly different during the operation or in the PACU among the groups (Fig 2).

**Fig 3:**
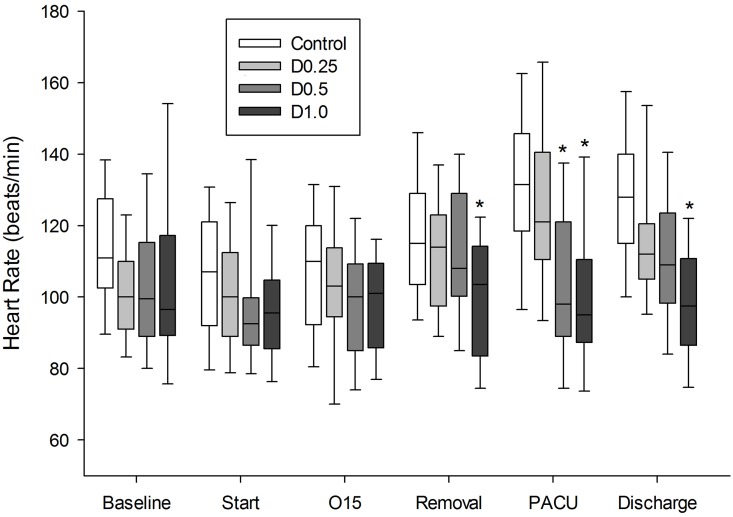
Change in heart rate during the study period. The horizontal line within the box is median heart rate during the study period. The edges of the box and the whiskers indicate 25–75% and 5–95% range respectively. The mean heart rate of D0.5 and D1.0 were significantly lower compared to control and D0.25 at PACU. * P < 0.05 vs. control and D0.25. Baseline = before induction; Start = start of operation; O15 = 15 min after start of operation, Removal = LMA removal, PACU = at admission to post-anesthesia care unit; Discharge = PACU discharge.

The mean heart rates of patients in group D1 were significantly lower than those of the controls and group D0.25 after the PACU arrival (Fig 3).

However, the values were within the norm and no patient needed atropine to correct bradycardia during the PACU stay.

The incidence of EA was 60, 48, 44, and 21% in the control, D0.25, D0.5, and D1 groups, respectively (*P* = 0.005); the incidence in group D1 was significantly lower than in the other groups (*P* < 0.05). The incidence of severe EA (EA score of 4) was 40, 16, 24, and 0% in the control, D0.25, D0.5, and D1 groups, respectively (*P* = 0.001); the incidence in group D1 was significantly lower than in the other groups (*P* < 0.05) (Table 3).

**Table 3:** Emergence agitation, oculocardiac reflex, postoperative pain, and fentanyl use.

Variable	Control	D 0.25	D 0.5	D 1.0	P-value
**N**	25	25	25	28	
**Agitation score ≥ 3**	15 (60%)	12 (48%)	11 (44%)	6 (21%)*^#^	0.005
**Agitation score 4**	10 (40%)	4 (16%)	6 (24%)	0 (0%)*^#^^§^	0.001
**PAED scale**	8.7 (4.1)	6.8 (4.9)	6.4 (4.5)	4.2 (4.0)*	0.005
**Fentanyl**	11 (44%)	8 (32%)	5 (20%)	3 (11%)*	0.037
**OCR**	9 (36%)	9 (41%)	9 (37.5%)	10 (37%)	0.988
**FLACC scale**	4.0 (2.1)	3.0 (2.0)	2.3 (1.9)*	1.4 (1.3) *^#^	<0.001

Values are mean (SD) or numbers (%).

* *P* < 0.05 *vs*. control,

^#^ *P* < 0.05 *vs*. D0.25,

^§^ *P* < 0.05 *vs*. D0.5.

PAED = Pediatric Anesthesia Emergence Delirium; OCR = oculocardiac reflex; FLACC = Face, Legs, Activity, Cry, Consolability.

Of the 44 patients who showed EA, 24 (54.5%) showed EA within 5 min of arriving in the PACU. The duration of EA was less than 10 min in most of the patients, except five control patients, two patients in group D0.25, and one patient in group D0.5. No patient in group D1 showed severe EA or EA for more than 10 min. The mean PAED scale differed significantly among the groups (*P* = 0.005), and a *post hoc* multiple comparison showed that the peak PAED scale for the D1 group was significantly lower than that of the controls (*P* < 0.05). The degree of postoperative pain, which was evaluated with the FLACC scale, also differed significantly among the groups (*P* < 0.001); the mean FLACC scale for groups D0.5 and D1 was significantly lower than that for the controls (*P* < 0.05). The PAED scales and FLACC pain scales were significantly correlated (Pearson’s correlation coefficient = 0.858). A total of 3 (3%) patients had preoperative agitation in the reception area and 16 (16%) on application of the facial mask, but the incidence did not differ among the groups (*P* = 0.689 and 0.209, respectively).

7 patients (1, 2, 2, and 2 in the group of control, D0.25, D0.5, and D1) experienced desaturation (pulse oximetry <95%) and recovered with encourage to breathe well. Only 2 patients (one person in each group of control and D0.5) showed POV without prophylaxis for postoperative nausea and vomiting and were treated with metoclopramide.

## Discussion

In our patients, dexmedetomidine reduced the incidence of EA without increasing the risk of the OCR in children undergoing strabismus surgery under desflurane anesthesia.

EA is a common post-anesthetic problem in the PACU that can potentially lead to self-injury and is a source of stress and labor for medical caregivers or parents. The reported incidence of EA is 10–80%, according to the scoring system, main anesthetic agent, and age of the patients studied [20–23]. Its etiology is unknown, but predictors include preschool age, the use of sevoflurane or desflurane, otorhinolaryngological or ophthalmological procedures, and postoperative pain [24, 25]. We limited the age of the patients recruited to 2–6 years, which is the age when patients are most vulnerable to EA.

Many analgesic drugs are effective at preventing EA, including ketamine, fentanyl, and α_2_-agonists. However, their potentiation of anesthesia or sedation, rather than their analgesic properties, is thought to be involved in the mechanism [20]. In this regard, dexmedetomidine, which is eight times more selective for α_2_-adrenoceptors than clonidine, is promising for preventing EA. Previous reports have shown that dexmedetomidine is effective at preventing EA, but the doses used ranged from 0.15 to 1 μg/kg, without any dose-response analysis [3–6, 26]. There are also meta-analyses showing that dexmedetomidine is a promising agent for the prevention of sevoflurane-related EA in children. We could infer that the effect would be similar in the prevention of desflurane-related EA in children but the most effective dose of dexmedetomidine for this purpose is not known [27, 28]. In this study, we found that dexmedetomidine at 1 μg/kg was more effective at preventing EA than dexmedetomidine at 0.25 or 0.5 μg/kg.

The most beneficial effects of dexmedetomidine are its sedative and analgesic effects, without respiratory depression. However, it might cause some hemodynamic changes. Dexmedetomidine at doses up to 2 μg/kg was well-tolerated in healthy adult volunteers, although it had a biphasic effect on blood pressure, with an initial increase and then a decline in blood pressure and a decrease in heart rate [29]. The hemodynamic effects of dexmedetomidine have also been evaluated in children and although it induced modest fluctuations in blood pressure and heart rate at doses around 2 μg/kg, these changes were usually within clinical norms, required no pharmacological intervention, and did not result in any adverse events [30]. We also observed relatively stable hemodynamics during the study period.

In our series, dexmedetomidine did not increase the incidence of the OCR, which is a trigemino-vagal reflex triggered by pressure on the globe, conjunctiva, and orbital structures and traction on the extraocular muscles. Conversely, dexmedetomidine decreases the heart rate mainly by decreasing the sympathetic tone and this coincided with reductions in the plasma noradrenalin and adrenalin levels [31]. Dexmedetomidine prevented epinephrine/halothane-induced ventricular tachycardia in an animal study, and this possible antiarrhythmic property is related to cerebral imidazoline receptors [32, 33]. Although dexmedetomidine decreased the heart rate during and after surgery, it had no effect on the incidence of the OCR. This result contrasts some previous reports who found a decreased incidence of the OCR with the use of dexmedetomidine during sevoflurane and ketamine anesthesia respectively and less drop of HR during dragging reflex under local anesthesia [12, 34, 35]. We cannot tell the reason of this discrepancy but a less marked analgesic potency of desflurane compared with its hypnotic action might have affected the results [36].

Our study has several limitations. First, both the PAED scales and FLACC pain scales have behavior components and it is difficult to distinguish real pain from EA exactly in young children. Indeed, in our data there was a significant correlation between the PAED scale and FLACC pain scale. This is in accordance with previous reports who indicated that pains are difficult to tell from symptoms of EA [26, 27]. Strabismus surgery in adults produced minimal postoperative pain which was controlled well with non-opioid drugs like oral acetaminophen, whether it was under general anesthesia or local ocular anesthesia, the reported pain scores in our study should be interpreted cautiously[37]. No painful examination such as MRI, children experienced EA after inhalation general anesthesia, and fentanyl was effective for EA after MRI [5]. Second, we measured hemodynamic variables only until discharge from the PACU. The hemodynamic effects of dexmedetomidine last around 300 min after an intravenous injection [29]. Although these data are from adults, caution is also needed with children if they show hemodynamic instability in the PACU. In our hospital, out-patient center where we sent the patients undergoing the strabismus surgery from PACU was close to PACU, and they monitored the patient’s vital sign, and complication like pain, POV, and desaturation. And there were no patients had hemodynamic instability after leaving the PACU. Third, the patients in our study had much less POV episodes compared to other study with strabismus surgery for children (2 patents- one person in each group of control and D0.5) without any other prophylaxis. Dexmedetomidine could have antiemetic effect for strabismus repairs of children [12, 27]. However it could not explain the patients in control group also showed minimal POV. We assumed the short surgical duration (its mean value and standard deviation were 28.4 and 7.0, respectively.) could be one factor [38].

## Conclusions

A single dose of dexmedetomidine reduced the incidence of EA in preschool children undergoing strabismus surgery under desflurane anesthesia without increasing the incidence of the OCR. Dexmedetomidine at 1 μg/kg was more effective at reducing EA compared to lower doses.

## Supporting Information

S1 CONSORT Checklist (DOC)

S1 File Statistics. (DOCX)

S1 Protocol Clinical study protocol in English. (DOCX)

S2 Protocol Clinical study protocol in Korean. (DOCX)
