# Supplementary material for: Dexmedetomidine Injection during Strabismus Surgery Reduces Emergence Agitation without Increasing the Oculocardiac Reflex in Children: A Randomized Controlled Trial
Source: PLoS One. 2016 Sep 12;11(9):e0162785. doi: 10.1371/journal.pone.0162785 (PMC5019399; doi:10.1371/journal.pone.0162785)
Supplement: S1 File — (DOCX) [file pone.0162785.s002.docx]

**Mean pain**

| **Descriptive Statistics** | | | | | | | | | | |
| --- | --- | --- | --- | --- | --- | --- | --- | --- | --- | --- |
|  | | | | | | | | | | |
|  | | N | Mean | SD | SE | 95% CI | | Min | Max | Component dispersion |
|  |  |  |  |  |  | lower | upper |  |  |  |
| 1 | | 25 | 4.0000 | 2.10214 | .42043 | 3.1323 | 4.8677 | .00 | 8.33 |  |
| 2 | | 25 | 3.0380 | 2.01303 | .40261 | 2.2071 | 3.8689 | .00 | 6.33 |  |
| 3 | | 25 | 2.3360 | 1.96204 | .39241 | 1.5261 | 3.1459 | .00 | 6.17 |  |
| 4 | | 28 | 1.4792 | 1.34670 | .25450 | .9570 | 2.0014 | .00 | 4.67 |  |
| whole | | 103 | 2.6773 | 2.06456 | .20343 | 2.2738 | 3.0808 | .00 | 8.33 |  |
| model | Fixed effect |  |  | 1.86588 | .18385 | 2.3125 | 3.0421 |  |  |  |
|  | Random effect |  |  |  | .54079 | .9563 | 4.3984 |  |  | 1.03199 |

| **ANOVA** | | | | | |
| --- | --- | --- | --- | --- | --- |
|  | | | | | |
|  | Sum of squares | Degree of freedom | Mean square | F | sig |
| Between group | 90.098 | 3 | 30.033 | 8.626 | .000 |
| Within group | 344.668 | 99 | 3.481 |  |  |
| whole | 434.766 | 102 |  |  |  |

| **Multiple Comparison** | | | | | | | |
| --- | --- | --- | --- | --- | --- | --- | --- |
|  | | | | | | | |
|  | (I) group | (J) group | Mean diff(I-J) | SE | Sig. probability | 95% CI | |
|  |  |  |  |  |  | lower | upper |
| Tukey HSD | 1 | 2 | .96200 | .52775 | .269 | -.4171 | 2.3411 |
|  |  | 3 | 1.66400^*^ | .52775 | .011 | .2849 | 3.0431 |
|  |  | 4 | 2.52083^*^ | .51342 | .000 | 1.1792 | 3.8625 |
|  | 2 | 1 | -.96200 | .52775 | .269 | -2.3411 | .4171 |
|  |  | 3 | .70200 | .52775 | .546 | -.6771 | 2.0811 |
|  |  | 4 | 1.55883^*^ | .51342 | .016 | .2172 | 2.9005 |
|  | 3 | 1 | -1.66400^*^ | .52775 | .011 | -3.0431 | -.2849 |
|  |  | 2 | -.70200 | .52775 | .546 | -2.0811 | .6771 |
|  |  | 4 | .85683 | .51342 | .346 | -.4848 | 2.1985 |
|  | 4 | 1 | -2.52083^*^ | .51342 | .000 | -3.8625 | -1.1792 |
|  |  | 2 | -1.55883^*^ | .51342 | .016 | -2.9005 | -.2172 |
|  |  | 3 | -.85683 | .51342 | .346 | -2.1985 | .4848 |
| LSD | 1 | 2 | .96200 | .52775 | .071 | -.0852 | 2.0092 |
|  |  | 3 | 1.66400^*^ | .52775 | .002 | .6168 | 2.7112 |
|  |  | 4 | 2.52083^*^ | .51342 | .000 | 1.5021 | 3.5396 |
|  | 2 | 1 | -.96200 | .52775 | .071 | -2.0092 | .0852 |
|  |  | 3 | .70200 | .52775 | .187 | -.3452 | 1.7492 |
|  |  | 4 | 1.55883^*^ | .51342 | .003 | .5401 | 2.5776 |
|  | 3 | 1 | -1.66400^*^ | .52775 | .002 | -2.7112 | -.6168 |
|  |  | 2 | -.70200 | .52775 | .187 | -1.7492 | .3452 |
|  |  | 4 | .85683 | .51342 | .098 | -.1619 | 1.8756 |
|  | 4 | 1 | -2.52083^*^ | .51342 | .000 | -3.5396 | -1.5021 |
|  |  | 2 | -1.55883^*^ | .51342 | .003 | -2.5776 | -.5401 |
|  |  | 3 | -.85683 | .51342 | .098 | -1.8756 | .1619 |
| Bonferroni | 1 | 2 | .96200 | .52775 | .428 | -.4589 | 2.3829 |
|  |  | 3 | 1.66400^*^ | .52775 | .013 | .2431 | 3.0849 |
|  |  | 4 | 2.52083^*^ | .51342 | .000 | 1.1386 | 3.9031 |
|  | 2 | 1 | -.96200 | .52775 | .428 | -2.3829 | .4589 |
|  |  | 3 | .70200 | .52775 | 1.000 | -.7189 | 2.1229 |
|  |  | 4 | 1.55883^*^ | .51342 | .018 | .1766 | 2.9411 |
|  | 3 | 1 | -1.66400^*^ | .52775 | .013 | -3.0849 | -.2431 |
|  |  | 2 | -.70200 | .52775 | 1.000 | -2.1229 | .7189 |
|  |  | 4 | .85683 | .51342 | .590 | -.5254 | 2.2391 |
|  | 4 | 1 | -2.52083^*^ | .51342 | .000 | -3.9031 | -1.1386 |
|  |  | 2 | -1.55883^*^ | .51342 | .018 | -2.9411 | -.1766 |
|  |  | 3 | -.85683 | .51342 | .590 | -2.2391 | .5254 |
| Dunnett t (both) | 2 | 1 | -.96200 | .52775 | .172 | -2.2200 | .2960 |
|  | 3 | 1 | -1.66400^*^ | .52775 | .006 | -2.9220 | -.4060 |
|  | 4 | 1 | -2.52083^*^ | .51342 | .000 | -3.7447 | -1.2970 |

|  | | | | | |
| --- | --- | --- | --- | --- | --- |
|  | group | N | Subsets of Level of significance = 0.05 | | |
|  |  |  | 1 | 2 | 3 |
| Student-Newman-Keuls^a,b^ | 4 | 28 | 1.4792 |  |  |
|  | 3 | 25 | 2.3360 | 2.3360 |  |
|  | 2 | 25 |  | 3.0380 | 3.0380 |
|  | 1 | 25 |  |  | 4.0000 |
|  | SIG |  | .103 | .181 | .068 |
| Tukey HSD^a,b^ | 4 | 28 | 1.4792 |  |  |
|  | 3 | 25 | 2.3360 | 2.3360 |  |
|  | 2 | 25 |  | 3.0380 | 3.0380 |
|  | 1 | 25 |  |  | 4.0000 |
|  | SIG |  | .358 | .535 | .257 |
| Tukey B^a,b^ | 4 | 28 | 1.4792 |  |  |
|  | 3 | 25 | 2.3360 | 2.3360 |  |
|  | 2 | 25 |  | 3.0380 | 3.0380 |
|  | 1 | 25 |  |  | 4.0000 |
|  | | | | | |
|  | | | | | |
|  | | | | | |

**PAED mean**

| **Descriptive Statistics** | | | | | | | | | | |
| --- | --- | --- | --- | --- | --- | --- | --- | --- | --- | --- |
|  | | | | | | | | | | |
|  | | N | Mean | SD | SE | 95% CI | | Min | Max | Component dispersion |
|  |  |  |  |  |  | 하한 | 상한 |  |  |  |
| 1 | | 25 | 8.7433 | 4.13410 | .82682 | 7.0369 | 10.4498 | .00 | 14.75 |  |
| 2 | | 25 | 6.8227 | 4.98274 | .99655 | 4.7659 | 8.8794 | .00 | 20.00 |  |
| 3 | | 25 | 6.4200 | 4.55061 | .91012 | 4.5416 | 8.2984 | .00 | 19.00 |  |
| 4 | | 28 | 4.2470 | 4.01358 | .75849 | 2.6907 | 5.8033 | .00 | 13.00 |  |
| whole | | 103 | 6.4909 | 4.65334 | .45851 | 5.5815 | 7.4004 | .00 | 20.00 |  |
| model | Fixed effect |  |  | 4.42442 | .43595 | 5.6259 | 7.3560 |  |  |  |
|  | Random effect |  |  |  | .93721 | 3.5083 | 9.4736 |  |  | 2.74624 |

| **Testing of homogeneity of dispersion** | | | |
| --- | --- | --- | --- |
|  | | | |
| Levene statistic | Degree of freedom1 | Degree of freedom2 | Sig probability |
| .384 | 3 | 99 | .765 |

| **ANOVA** | | | | | |
| --- | --- | --- | --- | --- | --- |
|  | | | | | |
|  | Sum of squares | Freedom | Mean square | F | Sig probability |
| Inter group | 270.693 | 3 | 90.231 | 4.609 | .005 |
| Within group | 1937.975 | 99 | 19.576 |  |  |
| whole | 2208.668 | 102 |  |  |  |

| **Multiple Comparison** | | | | | | | |
| --- | --- | --- | --- | --- | --- | --- | --- |
|  | | | | | | | |
|  | (I) group | (J) group | Mean diff(I-J) | SE | Sig probability | 95% CI | |
|  |  |  |  |  |  | lower | upper |
| Tukey HSD | 1 | 2 | 1.92067 | 1.25142 | .421 | -1.3495 | 5.1909 |
|  |  | 3 | 2.32333 | 1.25142 | .254 | -.9469 | 5.5935 |
|  |  | 4 | 4.49631^*^ | 1.21743 | .002 | 1.3149 | 7.6777 |
|  | 2 | 1 | -1.92067 | 1.25142 | .421 | -5.1909 | 1.3495 |
|  |  | 3 | .40267 | 1.25142 | .988 | -2.8675 | 3.6729 |
|  |  | 4 | 2.57564 | 1.21743 | .155 | -.6058 | 5.7571 |
|  | 3 | 1 | -2.32333 | 1.25142 | .254 | -5.5935 | .9469 |
|  |  | 2 | -.40267 | 1.25142 | .988 | -3.6729 | 2.8675 |
|  |  | 4 | 2.17298 | 1.21743 | .287 | -1.0084 | 5.3544 |
|  | 4 | 1 | -4.49631^*^ | 1.21743 | .002 | -7.6777 | -1.3149 |
|  |  | 2 | -2.57564 | 1.21743 | .155 | -5.7571 | .6058 |
|  |  | 3 | -2.17298 | 1.21743 | .287 | -5.3544 | 1.0084 |
| LSD | 1 | 2 | 1.92067 | 1.25142 | .128 | -.5624 | 4.4037 |
|  |  | 3 | 2.32333 | 1.25142 | .066 | -.1597 | 4.8064 |
|  |  | 4 | 4.49631^*^ | 1.21743 | .000 | 2.0807 | 6.9120 |
|  | 2 | 1 | -1.92067 | 1.25142 | .128 | -4.4037 | .5624 |
|  |  | 3 | .40267 | 1.25142 | .748 | -2.0804 | 2.8857 |
|  |  | 4 | 2.57564^*^ | 1.21743 | .037 | .1600 | 4.9913 |
|  | 3 | 1 | -2.32333 | 1.25142 | .066 | -4.8064 | .1597 |
|  |  | 2 | -.40267 | 1.25142 | .748 | -2.8857 | 2.0804 |
|  |  | 4 | 2.17298 | 1.21743 | .077 | -.2427 | 4.5886 |
|  | 4 | 1 | -4.49631^*^ | 1.21743 | .000 | -6.9120 | -2.0807 |
|  |  | 2 | -2.57564^*^ | 1.21743 | .037 | -4.9913 | -.1600 |
|  |  | 3 | -2.17298 | 1.21743 | .077 | -4.5886 | .2427 |
| Bonferroni | 1 | 2 | 1.92067 | 1.25142 | .768 | -1.4485 | 5.2899 |
|  |  | 3 | 2.32333 | 1.25142 | .398 | -1.0459 | 5.6925 |
|  |  | 4 | 4.49631^*^ | 1.21743 | .002 | 1.2186 | 7.7740 |
|  | 2 | 1 | -1.92067 | 1.25142 | .768 | -5.2899 | 1.4485 |
|  |  | 3 | .40267 | 1.25142 | 1.000 | -2.9665 | 3.7719 |
|  |  | 4 | 2.57564 | 1.21743 | .221 | -.7021 | 5.8533 |
|  | 3 | 1 | -2.32333 | 1.25142 | .398 | -5.6925 | 1.0459 |
|  |  | 2 | -.40267 | 1.25142 | 1.000 | -3.7719 | 2.9665 |
|  |  | 4 | 2.17298 | 1.21743 | .464 | -1.1047 | 5.4507 |
|  | 4 | 1 | -4.49631^*^ | 1.21743 | .002 | -7.7740 | -1.2186 |
|  |  | 2 | -2.57564 | 1.21743 | .221 | -5.8533 | .7021 |
|  |  | 3 | -2.17298 | 1.21743 | .464 | -5.4507 | 1.1047 |
| Dunnett t (both) | 2 | 1 | -1.92067 | 1.25142 | .292 | -4.9038 | 1.0624 |
|  | 3 | 1 | -2.32333 | 1.25142 | .161 | -5.3064 | .6598 |
|  | 4 | 1 | -4.49631^*^ | 1.21743 | .001 | -7.3984 | -1.5942 |

| **PAEDmean** | | | | |
| --- | --- | --- | --- | --- |
|  | group | N | Subsets of Level of significance = 0.05 | |
|  |  |  | 1 | 2 |
| Student-Newman-Keuls | 4 | 28 | 4.2470 |  |
|  | 3 | 25 | 6.4200 | 6.4200 |
|  | 2 | 25 | 6.8227 | 6.8227 |
|  | 1 | 25 |  | 8.7433 |
|  | Sig probability |  | .098 | .149 |
| Tukey HSD | 4 | 28 | 4.2470 |  |
|  | 3 | 25 | 6.4200 | 6.4200 |
|  | 2 | 25 | 6.8227 | 6.8227 |
|  | 1 | 25 |  | 8.7433 |
|  | Sig probability |  | .165 | .242 |
| Tukey B | 4 | 28 | 4.2470 |  |
|  | 3 | 25 | 6.4200 | 6.4200 |
|  | 2 | 25 | 6.8227 | 6.8227 |
|  | 1 | 25 |  | 8.7433 |
