# Supplementary material for: Dexmedetomidine Injection during Strabismus Surgery Reduces Emergence Agitation without Increasing the Oculocardiac Reflex in Children: A Randomized Controlled Trial
Source: PLoS One. 2016 Sep 12;11(9):e0162785. doi: 10.1371/journal.pone.0162785 (PMC5019399; doi:10.1371/journal.pone.0162785)
Supplement: S1 Protocol — (DOCX) [file pone.0162785.s003.docx]

|  |
| --- |
| The effect of dexmedetomidine on emergence agitation in children undergoing a surgery under desflurane anesthesia. |
|  |

| 2011.4.1.  Ah-young Oh |
| --- |

1. **Title**

The effect of dexmedetomidine on emergence agitation in children undergoing a surgery under desflurane anesthesia.

1. **Principal Investigator**

| Name | Ah-young Oh |
| --- | --- |
| Title | Professor |
| Affiliation | Seoul National University Bundang Hospital |
| Address | 82, Gumi-Ro 173 Beon-gil, Bundang-gu, Seongnam-si, Gyeonggi-do, 463-707,Korea |

1. **Purpose and Background**

Dexmedetomidine is a selective α-2-adrenergic agonist with sedative, anxiolytic, and analgesic effects with minimal respiratory depression. Its cardiovascular effect is modest, predictable, and dose-dependent. The use of dexmedetomidine in paediatric patients has been investigated extensively and it has been used as a premedication to reduce preoperative anxiety, as an adjunct to anaesthesia, for sedation in intensive care units, and for procedural sedation, without major complications.

Strabismus surgery is a very brief procedure. The oculocardiac reflex (OCR) is more frequent under propofol anaesthesia than under sevoflurane or desflurane anaesthesia. When using sevoflurane or desflurane, however, emergence agitation (EA) is a problem and strabismus surgery itself predisposes patients to developing EA. The perioperative use of dexmedetomidine reduces the incidence of EA. However, It also decrease heart rate and it is important to know how it affects OCR in anaesthetized patients with desflurane scheduled for strabismus surgery. In addition, the optimal dose of dexmedetomidine for this purpose has not been evaluated. Therefore, this study evaluated the effect of dexmedetomidine on EA and examined the dose of dexmedetomidine that best reduced EA without increasing the risk of the OCR.

1. **Name of drug and placebo**

Precedex 100mcg/1ml vial (Dexmedetomidine)

Placebo: Normal saline 0.9%

1. **Predicted duration for study**

1 year after approval of IRB

1. **Methods**
2. The study randomly allocated ASA class I pediatric patients, aged 2-6 years, undergoing elective strabismus surgery.
3. Random numbers were generated by a person not involved in the study using a computer-generated randomization code (Random Allocation Software, ver. 1.0; M. Saghaei, Isfahan University of Medical Sciences, Isfahan, Iran).
4. Using 4-point scale, preoperative agitation is evaluated in the reception area and again in the operating room before applying the mask to the patient.
5. In the operating room, the patient’s electrocardiograph, non-invasive arterial pressure, pulse oximetry, and end-tidal CO_2_ were monitored.
6. Without premedication, the patients were induced by the inhalation of sevoflurane and 60% N_2_O in the presence of one of their parents. After the loss of consciousness, an intravenous line was inserted and the study drug (saline or dexmedetomidine) was infused over 10 min.
7. The study drugs were prepared by an anesthesiologist who did not participate in the care of the patients to the same volume (10 ml) using saline.
8. After a few minutes of mask ventilation with 8% sevoflurane and 60% N_2_O, a laryngeal mask airway (LMA) was inserted and anesthesia was maintained with 8-10% desflurane and 60% N_2_O.
9. The desflurane concentration was adjusted to maintain the blood pressure and heart rate within 20% of the preoperative values. Neuromuscular blockers were not used. At the end of surgery, the LMA was removed in the operating room when the patient could breathe spontaneously and the time from the cessation of desflurane to removal of the LMA was recorded. The operations were done by a single experienced senior surgeon.
10. The lowest heart rate during the operation was recorded and a decrease in heart rate of > 20% from the baseline value, which was measured immediately before muscle we asked the surgeon to release the traction. Atropine at 0.01 mg/kg was used intravenously only for severe (< 60/min) or persistent bradycardia.
11. In the post-anesthesia care unit (PACU), EA and pain were evaluated continuously and recorded at 5-min intervals. EA was evaluated using a 4-point scale: 1 = calm; 2 = not calm, but could be calmed easily; 3 = not easily calmed, moderately agitated, or restless; and 4 = combative, excited, or disoriented. Patients with a score ≥ 3 were regarded as having EA. The Pediatric Anesthesia Emergence Delirium (PAED) scale was also checked and patients with a PAED score ≥ 10 were regarded as having EA. Pain was evaluated using the pediatric Face, Legs, Activity, Cry, and Consolability (FLACC) pain scale.
12. The experienced anesthesiologists evaluated the OCR, the severity of EA and pain using the scale described above, were blinded to the patients’ groups. They were standing at the patients’ bedside till they started to expressed the EA or pain, and treat them with fentanyl at 1 µg/kg immediately when they show severe agitation (EA score 4), or a FLACC pain score ≥ 6.
13. All of patients were encouraged to have 12.8mg/kg of acetaminophen suspension. Metoclopramide 0.1mg/kg IV were administered for patients with nausea and vomiting. Additionally 25mg of pethidine and 1mg/kg of ketorolac were prepared in case agitation or pain was relapsed after fentanyl’s effect disappeared.
14. All setting for anesthesia and perioperative treatment in the control group of this study was as same as routine children’s strabismus surgery before the study.
15. The durations of surgery and anesthesia, PACU stay time, and the time from stopping the inhalational agents to removing the LMA were also recorded. The events such as desaturation, bradycardia, nausea and vomiting, deep sedation in PACU were recorded.
    - Participants
      1. Indication : ASA class I, pediatric patients aged 2-6 years, undergoing elective strabismus surgery, outpatients
      2. Contraindication: lack of consent, known adverse effects to dexmedetomidine, mental retardation, developmental delay, or neurological or psychiatric illness that may associated with agitation (cerebral palsy, seizure, etc)
    - Sample size

Group sample sizes of 28 was calculated to detect a 40% reduction of the incidence of severe agitation with dexmedetomidine infusion from 57% in control group (normal saline infusion) with 0.05 of alpha and 0.80 of beta using Power Analysis and Sample Size software (PASS) 2013 (NCSS, LLC, Kaysville, Utah, USA) and was added with 10% of drop rate Allocation to the groups : randomization

- Random numbers were generated by a person not involved in the study using a computer-generated randomization code (Random Allocation Software, ver. 1.0; M. Saghaei, Isfahan University of Medical Sciences, Isfahan, Iran).
- Blindness :double blindness-> Subject, Investigator, Caregiver
- Dose, administration method of drug
  1. There are four groups accoding to dosage of dexmedetomidine. Dexmedetomidine 0.25 microg.kg(-1),(group D0.25), dexmedetomidine 0.5 microg.kg(-1),(Group D0.5), dexmedetomidine 1 microg.kg(-1) (Group D 1) and normal saline(group P) is administered ivs. for 10mins when we are inducing each patient.
- Other medication
  1. atropine 0.01 mg/kg: when patients have bradyarrhythmia
  2. fentanyl 1 ㎍/kg or other painkillers : when patients have agitation or need pain killer.
- We will record…

1. Age, weight, sex, height, the durations of surgery and anesthesia, time to LMA removal, recovery time, previous surgical history, and number of muscles operated
2. Complication : OCR, desaturation event, perioperative vomiting, or other complications
3. Pain: FLACC behavioral pain scale, fentanyl use
4. Emergence Agitation : 4-point scale, PAED scale

- Evaluation of primary outcome (EA)
  1. Evaluation of agitation will be done using Pediatric Anesthesia Emergence Delirium scale and 4 scale(a four-point scale: 1 _ calm; 2 _not calm but could be easily calmed; 3 _ not easily calmed, moderately agitated or restless; and 4 _ combative, xcited, or disoriented)
  2. Agitation : Pediatric Anesthesia Emergence Delirium scale>or =10, or 4 point scale> or =3
- PAED scale(Pediatric Anesthesia Emergence Delirium scale)

extremely not at all

| - 1. The child makes eye contact with the caregiver | - 0 1 2 3 4 |
| --- | --- |
| - 2. The child’s actions are purposeful | - 0 1 2 3 4 |
| - 3. The child is aware of his or her surroundings | - 0 1 2 3 4 |
| - 4. The child is restless | - 4 3 2 1 0 |
| - 5. The child is inconsolable | - 4 3 2 1 0 |

- Items 1,2,3 (reversed): 4 = not at all, 3 = just a little, 2 = quite a bit, 1 = very much, 0 = extremely
- Items 4,5: 0 = not at all, 1 = just a little, 2 = quite a bit, 3 = very much, 4 = extremely
  - 1. When patients get modified Aldrete score >10, they will discharge from PACU.
    2. We evaluated severity of pain using pediatric observational FLACC Pain
    3. (FLACC pain score ≥ 6 means severe pain)

| - **Categories** | - **scoring** | | |
| --- | --- | --- | --- |
|  | - 0 | - 1 | - 2 |
| - Face | - No particular expression or smile | - Occasional grimace or frown, withdrawn, disinterested | - Frequent to constant frown, clenched jaw, quivering chin |
| - Legs | - Normal position or relaxed | - Uneasy, restless, tense | - Arched, rigid, or jercking |
| - Activity | - Lying quietly, normal position, moves easily | - Squirming, shifting back and forth, tense | - Arched, rigid, or jerking |
| - Cry | - No cry (awake or asleep) | - Moans or whimpers, occasional complaint | - Crying steadily, screams or sobs, frequent complaints |
| - Consolability | - Content, relaxed | - Reassured by occasional touching, hugging, or being talked to, distractible | - Difficult to console or comfort |

- Safety
  1. When patients have bradycardia or hypotension, atropine 0.02mg /kg will be administrated ivs.
  2. Hypersensitivity, or critical events happened to patients, we will stop the drug for experiments, do emergent treatment, reveal what exactly the drug is ,and report about it to IRB.

.

- Statistical Analysis
- ANOVA :
  1. Age, weight, height, the durations of surgery and anesthesia, time to LMA removal, and recovery time were compared among the groups
  2. Heart rate and systolic blood pressure
- Pearson’s chi-square test
  1. The independence of sex, previous surgical history, and number of muscles operated
  2. trends in dichotomous data for agitation, fentanyl use, and OCR according to the dexmedetomidine dose (a linear-by-linear association and chi-square test.)
- the Kruskal-Wallis test and median tests
  1. preoperative agitation, PAED scale.
- P-values < 0.05 were considered significant.

1. **Study schedules**

For 7 months, enrollment, and data collection

For 2 month, review and analysis of data

For 3 month, writing paper

1. **References**

1. Yuen VM: Dexmedetomidine: perioperative applications in children. *Paediatr Anaesth* 2010, 20(3):256-264.

2. Young ET: Dexmedetomidine sedation in a pediatric cardiac patient scheduled for MRI. *Can J Anaesth* 2005, 52(7):730-732.

3. Chrysostomou C, Zeballos T: Use of dexmedetomidine in a pediatric heart transplant patient. *Pediatr Cardiol* 2005, 26(5):651-654.

4. Chrysostomou C, Di Filippo S, Manrique AM, Schmitt CG, Orr RA, Casta A, Suchoza E, Janosky J, Davis PJ, Munoz R: Use of dexmedetomidine in children after cardiac and thoracic surgery. *Pediatr Crit Care Med* 2006, 7(2):126-131.

5. Tobise F, Toyosmima Y, Kawana S: [Effect of dexmedetomidine on hemodynamics in pediatric patients following cardiac surgery]. *Masui* 2007, 56(4):409-413.

6. Chrysostomou C, Beerman L, Shiderly D, Berry D, Morell VO, Munoz R: Dexmedetomidine: a novel drug for the treatment of atrial and junctional tachyarrhythmias during the perioperative period for congenital cardiac surgery: a preliminary study. *Anesth Analg* 2008, 107(5):1514-1522.

7. Isik B, Arslan M, Tunga AD, Kurtipek O: Dexmedetomidine decreases emergence agitation in pediatric patients after sevoflurane anesthesia without surgery. *Paediatr Anaesth* 2006, 16(7):748-753.

8. Ibacache ME, Munoz HR, Brandes V, Morales AL: Single-dose dexmedetomidine reduces agitation after sevoflurane anesthesia in children. *Anesth Analg* 2004, 98(1):60-63, table of contents.

9. Olutoye OA, Glover CD, Diefenderfer JW, McGilberry M, Wyatt MM, Larrier DR, Friedman EM, Watcha MF: The effect of intraoperative dexmedetomidine on postoperative analgesia and sedation in pediatric patients undergoing tonsillectomy and adenoidectomy. *Anesth Analg* 2010, 111(2):490-495.
