# Supplementary material for: Dexmedetomidine Injection during Strabismus Surgery Reduces Emergence Agitation without Increasing the Oculocardiac Reflex in Children: A Randomized Controlled Trial
Source: PLoS One. 2016 Sep 12;11(9):e0162785. doi: 10.1371/journal.pone.0162785 (PMC5019399; doi:10.1371/journal.pone.0162785)
Supplement: S2 Protocol — (DOCX) [file pone.0162785.s004.docx]

| 분당 서울대병원 마취통증의학과 |
| --- |
| 수술 중 Dexmedetomidine 투여가 desflurane 흡입 마취 후 소아의 수술 후 섬망에 미치는 영향 |
| The effect of dexmedetomidine on emergence agitation in children undergoing a surgery under desflurane anesthesia. |

| 제출일시  2011.4.1.  오 아 영 |
| --- |

1. **연구 명칭**

국문: 수술 중 Dexmedetomidine 투여가 desflurane 흡입 마취 후 소아의 수술 후 섬망에 미치는 영향

영문: The effect of dexmedetomidine on emergence agitation in children undergoing a surgery under desflurane anesthesia.

1. **실시 기관 명 및 주소**

463-707 경기도 성남시 분당구 구미동 300번지 분당서울대학교병원 마취통증의학과

1. **연구 책임자 및 담당자, 공동연구자**

3.1 연구 책임자

오아영, 분당서울대학교병원 마취통증의학과 조교수

3.2 연구 담당자

오아영, 분당서울대학교병원 마취통증의학과 조교수

송인애, 분당서울대학교 병원 마취통증학과 촉탁의

3.3. 공동 연구자

송인애, 분당서울대학교병원 마취통증의학과 촉탁의

김진희, 분당 서울대학교병원 마취통증의학과 조교수

1. **연구의 목적 및 배경**

짧고 turn-over가 빠른 수술에 사용되는 마취제는 빠른 마취 유도 뿐 아니라, 마취로부터 빠른 회복이 필수적이며 마취시 심혈관계 억제가 적고 마취 관련 부작용이 적어야 한다. Sevoflurane이나 desflurane과 같은 최신 흡입마취제는 빠른 유도와 회복, 비교적 심혈관계 부작용이 적은 점 등의 이유로 수술 뿐 아니라 많은 소아 수술에 활용되어 왔다. 그러나, 이와 같은 최신 할로겐화 흡입마취제는 마취 회복 후 소아의 agitation 및 섬망의 원인으로 지목 받고 있다. 술 후 소아 agitation은 환자의 손상, 낙상의 원인이 되며 보건의료인력이 다수 동원되어도 억제하기 힘들다. 이 때 술 후 agitation을 줄이기 위해 fentanyl 1mcg/kg 정도를 사용하는 경우도 있으나, 간혹 호흡 억제, 심한 진정, 저산소증의 호흡기계 부작용이 나타나기도 한다. 이에 random double blind study를 통해 호흡 억제가 적고 진통 작용, 진정 작용도 있는dexmedetomidine을 술 중에 사용한 경우 이와 같은 agitation이 placebo를 투여했을 경우와 비교해서 효과적으로 감소하는지 살펴볼 것이다. Dexdetomidine은 중환자실에서의 진정 진통 목적 뿐 아니라[[1](#_ENREF_1)], 심질환이 있는 소아의 시술[[2](#_ENREF_2)], 및 심장 이식 수술[[3](#_ENREF_3)] 등에서 진정 [[4](#_ENREF_4)] 진통 및 활력 징후 안정[[2](#_ENREF_2), [5](#_ENREF_5)], 부정맥 감소 목적[[6](#_ENREF_6)]으로 연구되고 있으며, 그 밖에 MRI촬영시[[7](#_ENREF_7)] 또는 수술 및 마취후 agitation 방지[[8](#_ENREF_8), [9](#_ENREF_9)] 목적 외에도 진정 진통목적으로 널리 사용되고 있다. [[1](#_ENREF_1)]

1. **연구약의 코드명 (또는 주성분의 일반명), 원료약품 및 그 분량, 제형 등(대조약 포함)**

MDTIA(코드명), Precedex 100mcg/1ml vial

Dexmedetomidine

대조약: placebo: Normal saline 0.9%

1. **예상연구기간**

IRB 승인일로부터 1년간 (약 2012년 4월 30일까지)

1. **연구방법**
2. 연구방법개요
   - 1. 예정된 환자 중 2-6세 소아 환자를 대상으로 전향적 무작위 시험법 이중 맹검 연구를 시행할 예정이다.
     2. 술 전 환아 불안 정도를 평가한다.
     3. 난수표를 이용하여 dexmedetomidine 0.25 microg.kg(-1),(D0.25 군), dexmedetomidine 0.5 microg.kg(-1),(D0.5 군), dexmedetomidine 1 microg.kg(-1) (D 1군), 과 placebo 군(P군)으로 나누고 마취 유도시 세 군 모두 sevoflurane으로 마스크를 통해 용수 환기한다. 이중 맹검법으로 준비된 dexmedetomidine 0.25 microg.kg(-1), dexmedetomidine 0.5 microg.kg(-1), 1 microg.kg(-1) 또는 placebo인 normal saline을 투여한다.
     4. 마취 유도후 LMA 를 삽입하고 마취 유지는 desflurane과 N2O와 산소로 유지하며 근이완제는 사용하지 않는다. 마취 중 가스 농도(MAC)를 5분마다 기록한다. 마취 유도에 걸리는 시간을 기록하고 LMA 삽관시 기침, 호흡 정지, 기도 경련 등의 호흡기적인 합병증이나, 활력 징후가 baseline에 비해 30%이상 증가 또는 감소할 경우, 이를 기록하고 적절히 처치를 한다.
     5. 수술이 끝난 후 LMA를 제거하고 수술이 끝난 후 LMA 제거까지 걸린 시간 등, 눈을 뜰 때까지의 시간 등을 기록한다. 수술 및 마취 총 소요시간을 기록한다.
     6. Pediatric Anesthesia Emergence Delirium scale 및 4 scale(a four-point scale: 1 _ calm; 2 _ not calm but could be easily calmed; 3 _ not easily calmed, moderately agitated or restless; and 4 _ combative, xcited, or disoriented) 을 이용하여 agitation 의 정도를 퇴실할 때까지 5분마다 기록한다.
     7. modified Aldrete score, pediatric observational FLACC Pain Scale 및 퇴실까지의 시간을 기록한다. 수술중,후에 있을 수 있는 합병증 (호흡기계, 심혈관계 등)이 있을 시에는 이를 기록한다.
3. 피험자의 선정기준, 제외기준
   - 선정기준: 소아(2-6세) 당일 수술, ASA 1-2
   - 제외기준: lack of consent, known adverse effects to dexmedetomidine, mental retardation, developmental delay, or neurological or psychiatric illness that may associated with agitation (cerebral palsy, seizure, etc)
4. 목표 피험자의 수 및 산출 근거
   - 양측검정으로 p=0.05, 검정력을 0.8로 하였을 때, 참고논문의 자료를 바탕으로 severe agitation 발생율이 control 군과 dexmedetomidine 투여군이 각각 17%, 57%인 것을 바탕으로 탈락률 0.1을 감안하여 G*power 3.1.2.를 이용하여 각 군별로 29명씩(116명)이 필요하다고 결론을 내렸다.
5. 대조군의 설정
   - 대상 환자 중에 난수표를 이용하여 약물 투여군과 대조군으로 나뉜다.
6. 무작위배정
   - 난수표를 이용하여 환자군과 대조군으로 나눈다.
7. 눈가림법
   - 약물 준비자와 투여자 및 관찰자는 다른 사람으로 정한다. 투여자 및 관찰자는 해당 환자에게 투여되는 약물이 어떤 약물인지 알지 못한 상태에서 투여하고 관찰한다.
8. 시험약 투여량, 투여방법, 투여기간 및 설정사유
   - 1. 시험약 투여량: dexmedetomidine 0.25 microg.kg(-1),(D0.25 군), dexmedetomidine 0.5 microg.kg(-1),(D0.5 군), dexmedetomidine 1 microg.kg(-1) (D 1군)
     2. 투여방법: total 용량이 10ml가 되도록 N/S에 적절히 희석한 다음 iv.로 2분간 천천히 투여한다.,
     3. 투여량은 소아 대상 수술에서 진행한 비슷한 수술을 대상으로 한 논문을 참고하여 0.15-1.0micro.kg(-1) 중에 효과적으로 생각되는 용량을 설정하였다.
9. 대조약 사용시 그 선택사유
   - 1. Placebo군: normal saline으로 전체량은 동량으로 하여 효과는 없지만, dexmedetomidine과 구별이 어렵게 하였다.
10. 병용 요법
    - 1. atropine 0.01 mg/kg: 수술 중 서맥 발생시 치료목적
      2. fentanyl 1 ㎍/kg: 수술 후 통증 조절, 심한 각성섬망 치료

1. 관찰항목
   - 1. 환자의 나이, 키, 몸무게, ASA, 술전 이상소견, 수술장 입구 도착시의 환아의 불안 정도( 4-point scale), 수술 중 투여된 desflurane의 평균농도, 마취제 중단에서 눈뜸, LMA 발관, 각성섬망 발생까지의 시간, 각성섬망 지속시간, 총 수술 시간, 마취시간, 회복실 체류 시간, 마취 중 및 깨우는 도중, 회복실에서의 호흡기계, 심혈관계, vomiting 등의 event 기록.
     2. 수술 후 통증: FLACC behavioral pain scale
     3. 각성섬망: 4-point scale, PAED scale
2. 효과평가변수, 평가방법 및 해석방법
   - 1. Pediatric Anesthesia Emergence Delirium scale 및 4 scale(a four-point scale: 1 _ calm; 2 _

not calm but could be easily calmed; 3 _ not easily calmed, moderately agitated or restless; and 4 _ combative, xcited, or disoriented) 을 이용하여 agitation 의 정도를 평가하고 Pediatric Anesthesia Emergence Delirium scale 이 10점 이상, 4 point scale이 3이상인 경우를 agitation이 있다고 정의하고 2이하의 경우는 no agiation으로 정의하여 agitation의 발생율 간에 두 군 간에 통계적인 차이가 있는지 서로 비교한다.

- PAED scale(Pediatric Anesthesia Emergence Delirium scale)

extremely not at all

| 1. The child makes eye contact with the caregiver | 0 1 2 3 4 |
| --- | --- |
| 2. The child’s actions are purposeful | 0 1 2 3 4 |
| 3. The child is aware of his or her surroundings | 0 1 2 3 4 |
| 4. The child is restless | 4 3 2 1 0 |
| 5. The child is inconsolable | 4 3 2 1 0 |

- - Items 1,2,3 (reversed): 4 = not at all, 3 = just a little, 2 = quite a bit, 1 = very much, 0 = extremely
  - Items 4,5: 0 = not at all, 1 = just a little, 2 = quite a bit, 3 = very much, 4 = extremely
    1. modified Aldrete score >10일 때, 퇴실을 결정한다.
    2. pediatric observational FLACC Pain Scale 으로 통증 정도를 평가하여 6이상의 경우 심한 통증으로 정의한다.

| **categories** | **scoring** | | |
| --- | --- | --- | --- |
|  | 0 | 1 | 2 |
| Face | No particular expression or smile | Occasional grimace or frown,withdrawn, disinterested | Frequent to constant frown, clenched jaw, quivering chin |
| Legs | Normal position or relaxed | Uneasy, restless, tense | Arched, rigid, or jercking |
| Activity | Lying quietly, normal position, moves easily | Squirming, shifting back and forth, tense | Arched, rigid, or jerking |
| Cry | No cry (awake or asleep) | Moans or whimpers, occasional complaint | Crying steadily, screams or sobs, frequent complaints |
| Consolability | Content, relaxed | Reassured by occasional touching, hugging, or being talked to, distractible | Difficult to console or comfort |

1. 안전성평가기준, 평가방법 및 보고방법:

서맥, 저혈압 발생시 atropine 0.02mg /kg ivs. 투여한다. 과민반응 등 예기치 않은 부작용이 생길 경우, 즉시 주입을 중단하고 증상에 맞는 치료를 한 후 IRB위원회에 보고한다.

.

1. 통계분석 원칙 및 방법
   - one way ANOVA 및 pearson 카이 제곱 검정(p<0.05)
2. **피험자에 대한 안전성의 배려(시험중지, 심각한 부작용에 대한 대처 사항 등)**

- 마취에 따른 일반적인 부작용이 나타날 경우, 일반적인 처치를 한다. 저혈압, 서맥시 baseline 환자의 활력징후의 30%이상 변동한 경우, 각각 ephedrine과 atropine 0.02mg/kg를 투여하고 고혈압, 빈맥 시에는 마취 심도를 높힌다.

-약물 주입시 과민반응이 나타나면 즉시 주입을 중지하고 과민반응의 증상에 따라 치료를 시작하며 시험을 중단한다. (IRB 위원회에 보고한다.)

1. **연구수행일정표**

IRB 통과 이후 7개월 동안 임상시험

2개월동안 자료 정리 및 리뷰, 추가 시험 실시

3개월간 논문 작성 및 발표

1. **참고문헌**

**1. Yuen VM: Dexmedetomidine: perioperative applications in children. *Paediatr Anaesth* 2010, 20(3):256-264.**

**2. Young ET: Dexmedetomidine sedation in a pediatric cardiac patient scheduled for MRI. *Can J Anaesth* 2005, 52(7):730-732.**

**3. Chrysostomou C, Zeballos T: Use of dexmedetomidine in a pediatric heart transplant patient. *Pediatr Cardiol* 2005, 26(5):651-654.**

**4. Chrysostomou C, Di Filippo S, Manrique AM, Schmitt CG, Orr RA, Casta A, Suchoza E, Janosky J, Davis PJ, Munoz R: Use of dexmedetomidine in children after cardiac and thoracic surgery. *Pediatr Crit Care Med* 2006, 7(2):126-131.**

**5. Tobise F, Toyosmima Y, Kawana S: [Effect of dexmedetomidine on hemodynamics in pediatric patients following cardiac surgery]. *Masui* 2007, 56(4):409-413.**

**6. Chrysostomou C, Beerman L, Shiderly D, Berry D, Morell VO, Munoz R: Dexmedetomidine: a novel drug for the treatment of atrial and junctional tachyarrhythmias during the perioperative period for congenital cardiac surgery: a preliminary study. *Anesth Analg* 2008, 107(5):1514-1522.**

**7. Isik B, Arslan M, Tunga AD, Kurtipek O: Dexmedetomidine decreases emergence agitation in pediatric patients after sevoflurane anesthesia without surgery. *Paediatr Anaesth* 2006, 16(7):748-753.**

**8. Ibacache ME, Munoz HR, Brandes V, Morales AL: Single-dose dexmedetomidine reduces agitation after sevoflurane anesthesia in children. *Anesth Analg* 2004, 98(1):60-63, table of contents.**

**9. Olutoye OA, Glover CD, Diefenderfer JW, McGilberry M, Wyatt MM, Larrier DR, Friedman EM, Watcha MF: The effect of intraoperative dexmedetomidine on postoperative analgesia and sedation in pediatric patients undergoing tonsillectomy and adenoidectomy. *Anesth Analg* 2010, 111(2):490-495.**
